# Effect of a 7-Day and 14-Day Antibiotic Time-Out Reminder Alert (TORA) on Inpatient Piperacillin-Tazobactam Use: an interrupted time-series study

**DOI:** 10.1017/ash.2026.10899

**Published:** 2026-10-05

**Authors:** Yuhei Suzuki, Ryo Yamaguchi, Takehito Yamamoto, Sohei Harada, Takehiro Tanaka, Satoshi Kitaura, Shinya Matsumoto, Ryu Yoneda, Takeya Tsutsumi, Tappei Takada

**Affiliations:** 1 Department of Pharmacy, The University of Tokyo Hospital, Tokyo, Japan; 2 Department of Microbiology and Infectious Diseases, Toho University School of Medicine, Tokyo, Japan; 3 Department of Infectious Diseases, The University of Tokyo Hospital, Tokyo, Japan; 4 Department of Infection Control and Prevention, The University of Tokyo Hospital, Tokyo, Japan

## Abstract

**Objective:**

To evaluate whether a low-burden antimicrobial stewardship intervention, the 7-Day and 14-Day Antibiotic Time-Out Reminder Alert (TORA), was associated with changes in inpatient piperacillin-tazobactam (PIPC/TAZ) use.

**Design:**

Quasi-experimental interrupted time-series study.

**Setting:**

A tertiary-care teaching hospital in Japan.

**Participants:**

Inpatients in targeted departments, excluding hematology and transplant surgery.

**Methods:**

TORA was introduced on September 1, 2021, using day-7 and day-14 reminders for ongoing PIPC/TAZ therapy in the electronic medical record. The primary outcome was monthly PIPC/TAZ use as days of therapy (DOT) per 100 patient-days. Segmented regression analysis used generalized least squares with a first-order autoregressive correlation structure and month fixed effects. Balancing outcomes included carbapenems, antipseudomonal cephalosporins, and quinolones. Secondary outcomes included treatment episodes lasting ≥7 and ≥14 days and 30- and 90-day mortality.

**Results:**

TORA was associated with an immediate reduction in PIPC/TAZ use (level change, −1.021 DOT/100 patient-days; 95% CI, −1.783 to −0.259; *P* = .010) but no significant change in postintervention slope (−0.007 DOT/100 patient-days per month; 95% CI, −0.044 to 0.031; *P* = .724). Carbapenem and quinolone use showed no significant immediate or slope changes, whereas antipseudomonal cephalosporin use decreased immediately without slope changes. The proportions of treatment episodes lasting ≥7 and ≥14 days decreased significantly. Thirty- and 90-day mortality did not differ significantly before and after implementation.

**Conclusions:**

A low-burden TORA strategy was associated with an immediate reduction in inpatient PIPC/TAZ use and may offer a pragmatic stewardship approach where personnel-intensive interventions are difficult to sustain.

## Introduction

Antimicrobial stewardship programs (ASPs) optimize antibiotic use in hospitalized patients, but core interventions such as prospective audit and feedback (PAF) and preauthorization are resource intensive and may be difficult to sustain across all departments.^1,2^ When personnel are limited, stewardship targets must therefore be prioritized.

Piperacillin-tazobactam (PIPC/TAZ) is widely used for empirical therapy.^3^ Inappropriate empirical use and unnecessary continuation are common.^4,5^ Internal benchmarking using the Japan Surveillance for Infection Prevention and Healthcare Epidemiology (J-SIPHE) identified PIPC/TAZ use as a local stewardship priority.^6^ Therefore, optimizing PIPC/TAZ use became one of the institutional stewardship priorities at our hospital.

Antibiotic time-out is a relatively low-burden, prescriber-centered strategy that prompts reassessment of antibiotic indication, microbiologic data, and opportunities for discontinuation or de-escalation after empirical therapy begins.^1,7^ Prior studies suggest potential benefits including improved de-escalation, shorter treatment duration, and reduced use of selected target agents.^8–10^

However, effectiveness varies across settings and intervention formats.^11–14^ Most studies have evaluated time-outs at 48–72 hours, when accumulating microbiologic and clinical information supports reassessment of empirical therapy and de-escalation.^7,9,10,12^ Later decision points such as day 7 and day 14 instead address the need for continued broad-spectrum therapy and treatment duration,^15^ particularly when earlier de-escalation is difficult because of clinical instability or diagnostic uncertainty. Evidence for simple reminder-based approaches at these later milestones remains limited. This gap is relevant to ASPs operating with limited personnel resources.^2^

Therefore, we evaluated whether a simple, low-burden 7-Day and 14-Day Antibiotic Time-Out Reminder Alert (TORA), delivered through patient-level electronic medical record (EMR) message-board reminders and standardized documentation for ongoing PIPC/TAZ therapy, was associated with inpatient PIPC/TAZ use in targeted departments.

## Methods

### Study design and setting

We conducted a quasi-experimental study using interrupted time-series (ITS) analysis of monthly aggregated antimicrobial-use data at The University of Tokyo Hospital (UTH), Tokyo, Japan. UTH is a 1,208-bed tertiary-care teaching hospital. Physicians could consult infectious diseases specialists at any time. Monthly inpatient antimicrobial-use data were obtained from our institution’s J-SIPHE submissions and expressed as days of therapy (DOT) per 100 patient-days.^16,17^ The preintervention period was September 2018 through August 2021, and the postintervention period was September 2021 through August 2024.

### ASP activities and TORA intervention

Throughout the study period, ASP pharmacists conducted weekday PAF for all patients receiving anti-MRSA agents or carbapenems and reviewed patients with positive blood cultures. Carbapenems required notification after initiation but not preauthorization; PIPC/TAZ, antipseudomonal cephalosporins, and quinolones required no preauthorization. Aggregate antimicrobial-use trends were reviewed every 2 months by the Infection Control Committee. ASP pharmacists also identified via the EMR patients receiving antipseudomonal agents for >14 consecutive days and discussed them weekly with ASP physicians. When intervention was warranted, recommendations were communicated directly to treating physicians by telephone or face-to-face discussion and documented in the EMR. These activities remained unchanged throughout the study period. After TORA implementation, PIPC/TAZ courses >14 days remained subject to this review in addition to the day-14 reminder.

Beginning in September 2021, we introduced TORA targeting prolonged PIPC/TAZ treatment in all departments except hematology and transplant surgery, where antipseudomonal therapy was often indicated and treatment decisions frequently required individualized consideration.^18,19^ TORA consisted of a standardized EMR note and a patient-level message-board reminder for PIPC/TAZ therapy exceeding 7 or 14 consecutive days; the reminder remained visible when the chart was opened until expiration. TORA itself did not routinely involve telephone or face-to-face communication with prescribers. Standardized day-7 and day-14 texts are provided in Supplementary Appendix 1. The texts reflected published guidance on usual treatment durations and situations requiring longer or individualized therapy and encouraged infectious diseases consultation when the stop date was uncertain.^20,21^

Potential intervention candidates were identified on weekdays by automatically extracting current PIPC/TAZ users and consecutive treatment days from the EMR, followed by automated filtering with an Excel macro to exclude hematology and transplant surgery and to identify treatment episodes exceeding 7 or 14 consecutive days. Final eligibility was confirmed by manual chart review, and episodes with a formal infectious diseases consultation order already in place at the relevant threshold (day 7 or day 14) were excluded from reminder placement. Treatment days were counted as consecutive days from the first day of administration (day 1). If PIPC/TAZ was discontinued and later restarted, the restarted course was treated as a new treatment episode. A day-7 reminder was posted once when a treatment episode exceeded 7 consecutive days. If therapy subsequently exceeded 14 consecutive days, an additional day-14 reminder was posted once. For the message-board reminder, visibility was preset to expire 7 days after posting. When thresholds were reached on weekends or holidays, reminders were posted on the next business day. The intervention was delivered on weekdays by 3 ASP pharmacists who rotated responsibility. The entire workflow—from EMR extraction and Excel-based screening to manual chart review and posting of reminders—typically required approximately 15 minutes per weekday. The intervention was approved by the hospital Infection Control Committee and announced prior to implementation at a hospitalwide infection control liaison meeting attended by departmental representatives. During the analytic period, no major PIPC/TAZ-specific operational changes likely to substantially confound the intervention effect were identified, including PIPC/TAZ supply restrictions, formulary changes, or changes in administration-restriction rules.

### Outcomes

The primary outcome was monthly inpatient PIPC/TAZ use, expressed as DOT per 100 patient-days, in the targeted departments. Carbapenems, antipseudomonal cephalosporins, and quinolones were assessed as balancing outcomes to examine potential substitution of PIPC/TAZ with other broad-spectrum intravenous antipseudomonal agents, using monthly DOT per 100 patient-days in the targeted departments. Use of aztreonam, piperacillin alone, and aminoglycosides was additionally examined descriptively as potential substitutable agents. Carbapenems comprised doripenem, biapenem, meropenem, panipenem/betamipron, imipenem/cilastatin, and imipenem/cilastatin/relebactam. Antipseudomonal cephalosporins comprised cefoperazone, ceftazidime, cefoperazone/sulbactam, ceftazidime/avibactam, cefepime, cefozopran, cefpirome, and ceftolozane/tazobactam. Quinolones comprised ciprofloxacin, pazufloxacin, and levofloxacin. Because the monthly J-SIPHE-based aggregated data did not allow exclusion of cases with infectious diseases consultation, such cases remained included in the department-level utilization outcomes.

Secondary outcomes were assessed as descriptive prepost comparisons at two levels of analysis. At the treatment-episode level, we evaluated the proportion of PIPC/TAZ treatment episodes lasting ≥7 consecutive days and ≥14 consecutive days. At the patient level, we evaluated 30-day and 90-day mortality among inpatients who received PIPC/TAZ during the study period, with follow-up starting on the date of PIPC/TAZ initiation. Mortality was ascertained from the EMR, including routinely recorded follow-up information after discharge when available. We also evaluated 30-day mortality in patients who had at least one positive blood culture within 48 hours before PIPC/TAZ initiation to assess clinical outcomes in a higher-risk subgroup with microbiologically documented bloodstream infection. In this subgroup, day 0 was defined as the date of blood culture collection.

### Statistical analysis

Segmented regression models for ITS analyses were fitted using generalized least squares. Separate models were fitted for PIPC/TAZ, carbapenems, antipseudomonal cephalosporins, and quinolones. The model specification was:

$$\begin{array}{rl} Y_{t} & =\beta_{0}+\beta_{1}{time}_{t}+\beta_{2}{post}_{t}+\beta_{3}{time\_after}_{t}\\ & +\sum_{m=2}^{12}\gamma_{m}{Month}_{m,t}+\varepsilon_{t} \end{array}$$

where *Y_t_* was the monthly outcome; time*_t_* was a continuous monthly variable with September 2018 coded as 0; post*_t_* was an indicator for the postintervention period (1 from September 2021 onward, 0 otherwise); time_after*_t_* was 0 before intervention and in September 2021, then increased by 1 each month (October 2021 = 1); and ϵ*_t_* was the error term. Month*_m_*,*_t_* was 1 for calendar month m and 0 otherwise, with January as the reference. Calendar-month fixed effects accounted for seasonality; a first-order autoregressive [AR(1)] error structure accounted for serial autocorrelation. For PIPC/TAZ, normalized residual diagnostics supported AR(1), showing reduced lag-1 autocorrelation and a nonsignificant Ljung–Box test at lag 12. The coefficient for post*_t_* represented the immediate level change at intervention implementation, and the coefficient for time_after*_t_* represented the change in postintervention slope. Treatment-episode-level and patient-level secondary outcomes, including the blood-culture-positive subgroup analysis, were compared between periods using Fisher’s exact test. There were no missing data for the monthly aggregated ITS outcomes. All tests were two-sided, and *P* < .05 was considered statistically significant. Analyses were performed using R version 4.3.1 (R Foundation for Statistical Computing, Vienna, Austria).

### Ethics

This study was approved by the institutional review board of The University of Tokyo Hospital (approval no. 2529-[16]). The requirement for informed consent was waived because of the retrospective nature of the study.

## Results

### Intervention implementation

TORA was implemented on September 1, 2021, in all departments except hematology and transplant surgery. During the postintervention period, 1,716 reminders were posted, including 1,475 day-7 reminders and 241 day-14 reminders, corresponding to a mean of 2.3 reminders per weekday.

### Monthly antibiotic use

**Figure 1.**
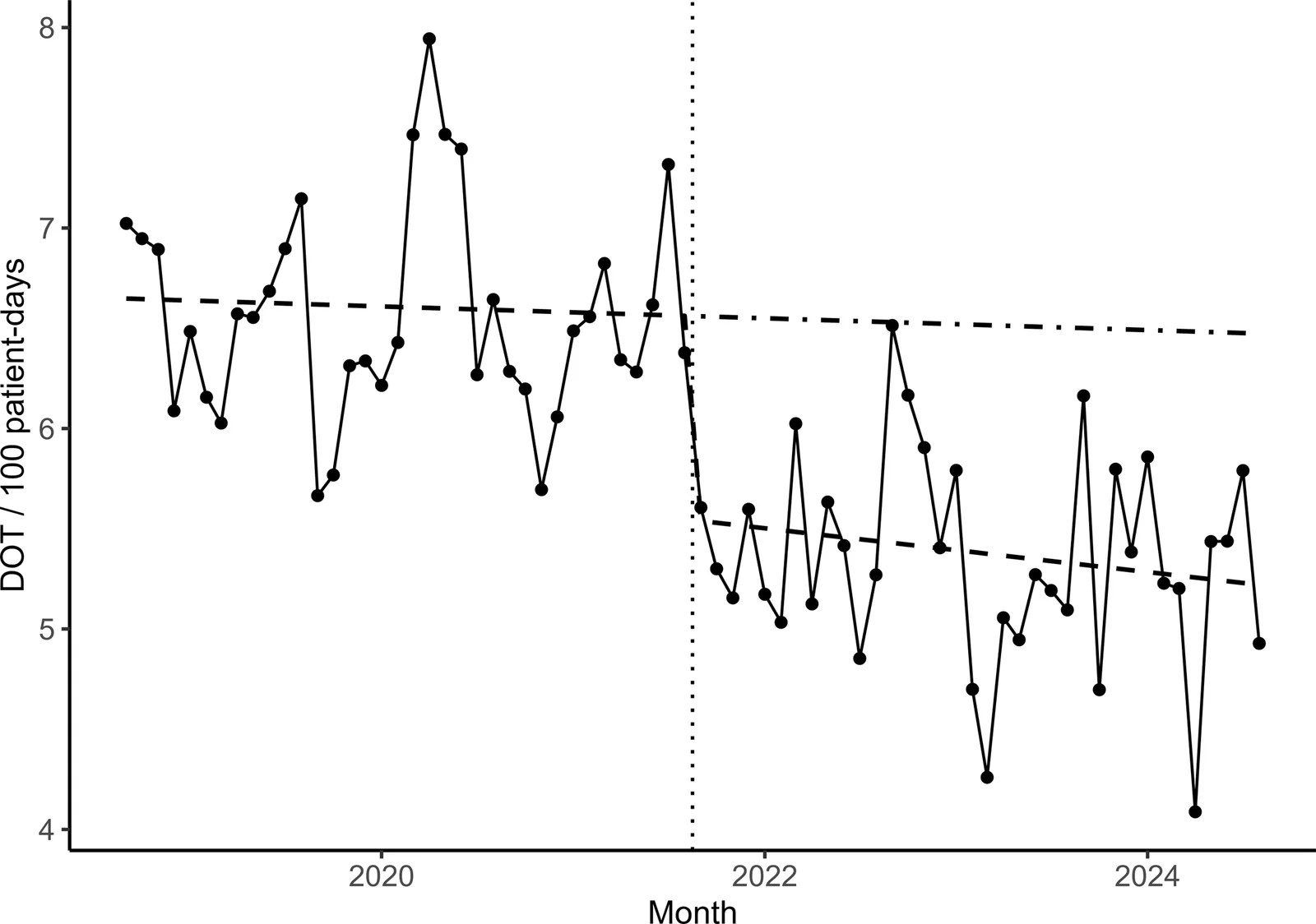
Monthly inpatient PIPC/TAZ use in the targeted departments is shown as days of therapy (DOT) per 100 patient-days from September 2018 through August 2024. The vertical dotted line indicates the boundary between the preintervention and postintervention periods, corresponding to TORA implementation on September 1, 2021; September 2021 was the first postintervention month. TORA consisted of standardized day-7 and day-14 reminders documented in the EMR and displayed as patient-level message-board reminders within the EMR for ongoing PIPC/TAZ therapy. Circles connected by the solid line represent observed monthly values. The dashed line represents the fitted segmented trend. The dot-dash line represents the counterfactual trajectory projected from the preintervention trend. DOT, days of therapy; PIPC/TAZ, piperacillin-tazobactam; TORA, 7-Day and 14-Day Antibiotic Time-Out Reminder Alert.

Figure 1 shows monthly inpatient PIPC/TAZ use in the targeted departments from September 2018 through August 2024. In segmented regression analysis, TORA implementation was associated with an immediate reduction in PIPC/TAZ use (estimate, −1.021 DOT per 100 patient-days; 95% CI, −1.783 to −0.259; *P* = .010), whereas the change in postintervention slope was not significant (estimate, −0.007; 95% CI, −0.044 to 0.031; *P* = .724) (Table 1). To assess possible therapeutic substitution to other antipseudomonal agents, we also examined carbapenems, antipseudomonal cephalosporins, and quinolones as balancing antibiotic-use outcomes. Carbapenem use showed no significant immediate change (estimate, 0.109; 95% CI, −0.354 to 0.572; *P* = .639) and no significant slope change (estimate, −0.010; 95% CI, −0.033 to 0.013; *P* = .400). Antipseudomonal cephalosporin use showed a significant immediate reduction (estimate, −0.480; 95% CI, −0.830 to −0.129; *P* = .008), but no significant slope change (estimate, 0.012; 95% CI, −0.005 to 0.029; *P* = .160). Quinolone use showed neither a significant immediate change (estimate, −0.012; 95% CI, −0.252 to 0.228; *P* = .921) nor a significant slope change (estimate, −0.008; 95% CI, −0.020 to 0.004; *P* = .207) (Table 1). Aztreonam and aminoglycoside use remained low and similar between periods, whereas piperacillin-alone use increased from 0.023 to 0.056 DOT per 100 patient-days but remained minimal relative to PIPC/TAZ use.

**Table 1.** Estimated changes in monthly inpatient PIPC/TAZ use and balancing antibiotic-use outcomes after implementation of the 7-Day and 14-Day Antibiotic Time-Out Reminder Alert (TORA) Table 1 long description.

Drug group	Variable	Estimate (95% CI)	*P* value
PIPC/TAZ	Immediate change	−1.021 (−1.783 to −0.259)	.010
	Slope change	−0.007 (−0.044 to 0.031)	.724
Carbapenems	Immediate change	0.109 (−0.354 to 0.572)	.639
	Slope change	−0.010 (−0.033 to 0.013)	.400
Antipseudomonal cephalosporins	Immediate change	−0.480 (−0.830 to −0.129)	.008
	Slope change	0.012 (−0.005 to 0.029)	.160
Quinolones	Immediate change	−0.012 (−0.252 to 0.228)	.921
	Slope change	−0.008 (−0.020 to 0.004)	.207

CI, confidence interval; DOT, days of therapy; PIPC/TAZ, piperacillin-tazobactam; TORA, 7-Day and 14-Day Antibiotic Time-Out Reminder Alert.

Note. Estimates were obtained from separate single-group interrupted time-series models of monthly inpatient antibiotic use (DOT per 100 patient-days) in targeted departments from September 2018 through August 2024. TORA was implemented on September 1, 2021. Models were fitted using generalized least squares with an AR(1) error structure and calendar-month fixed effects. “Immediate change” indicates the level change at implementation, and “slope change” indicates the change in postimplementation monthly trend. Carbapenems comprised doripenem, biapenem, meropenem, panipenem/betamipron, imipenem/cilastatin, and imipenem/cilastatin/relebactam. Antipseudomonal cephalosporins comprised cefoperazone, ceftazidime, cefoperazone/sulbactam, ceftazidime/avibactam, cefepime, cefozopran, cefpirome, and ceftolozane/tazobactam. Quinolones comprised ciprofloxacin, pazufloxacin, and levofloxacin.

### Secondary outcomes

Secondary outcomes are shown in Table 2. At the treatment-episode level, the proportion of PIPC/TAZ treatment episodes lasting ≥7 consecutive days decreased from 54.8% (3,582/6,542) in the preintervention period to 51.1% (2,836/5,555) in the postintervention period (*P* < .001). The proportion of episodes lasting ≥14 consecutive days also decreased from 15.0% (984/6,542) to 10.3% (573/5,555) (*P* < .001). At the patient level, 30-day mortality from PIPC/TAZ initiation was 7.7% (337/4,369) in the preintervention period and 7.8% (285/3,648) in the postintervention period (*P* = .87). Ninety-day mortality was 10.2% (447/4,369) before the intervention and 10.1% (368/3,648) after the intervention (*P* = .85). Among patients with at least 1 positive blood culture within 48 hours before PIPC/TAZ initiation, 30-day mortality from the date of blood culture collection was 9.5% (50/525) in the preintervention period and 12.1% (44/364) in the postintervention period (*P* = .23) (Table 2).

**Table 2.** Secondary outcomes before and after implementation of the 7-Day and 14-Day Antibiotic Time-Out Reminder Alert (TORA) Table 2 long description.

Outcome	Preintervention	Postintervention	*P* value
**Treatment-episode-level outcomes**			
PIPC/TAZ treatment episodes, No.	6,542	5,555	
Episodes lasting ≥7 consecutive days, n (%)	3,582 (54.8)	2,836 (51.1)	<.001
Episodes lasting ≥14 consecutive days, n (%)	984 (15.0)	573 (10.3)	<.001
**Patient-level outcomes**			
Patients receiving PIPC/TAZ, No.	4,369	3,648	
30-day mortality from PIPC/TAZ initiation, n (%)	337 (7.7)	285 (7.8)	.87
90-day mortality from PIPC/TAZ initiation, n (%)	447 (10.2)	368 (10.1)	.85
**Blood-culture-positive subgroup**			
Patients with ≥1 positive blood culture within 48 hours before PIPC/TAZ initiation, No.	525	364	
30-day mortality from blood culture collection, n (%)	50 (9.5)	44 (12.1)	.23

PIPC/TAZ, piperacillin-tazobactam; TORA, 7-Day and 14-Day Antibiotic Time-Out Reminder Alert.

Note. Preintervention period, September 2018 through August 2021; postintervention period, September 2021 through August 2024. Treatment-episode-level and patient-level secondary outcomes were compared between periods using Fisher’s exact test. In the blood-culture-positive subgroup, day 0 was defined as the date of blood culture collection. For the blood-culture-positive subgroup analysis, duplicate patient IDs and patients transferred to another hospital were excluded.

## Discussion

In this interrupted time-series study, implementation of a simple, low-burden TORA intervention was associated with an immediate reduction in inpatient PIPC/TAZ use in targeted departments. The proportions of PIPC/TAZ treatment episodes lasting ≥7 and ≥14 days were also lower after implementation. In contrast, we did not observe evidence of increased carbapenem or quinolone use after implementation, and 30- and 90-day mortality did not differ significantly between periods.

PIPC/TAZ use decreased immediately after TORA implementation, with no significant slope change. Heightened attention to a newly introduced reminder may have contributed to the immediate response, although message readership and prescriber responses were not measured. Conversely, TORA relied on unidirectional EMR reminders; unread or unacted-upon messages may have attenuated the immediate effect. Responsiveness may also have changed over time, including through alert fatigue, potentially influencing the subsequent trend. These mechanisms could not be directly evaluated. The observed pattern is compatible with a sustained level reduction, but the nonsignificant slope change alone cannot establish stable responsiveness or alert fatigue. Prior studies likewise suggest that time-outs may improve prescribing processes, while effects on aggregate antibiotic use vary across settings.^9,11,22^

The balancing antibiotic-use outcomes did not suggest major therapeutic substitution to carbapenems or quinolones. Antipseudomonal cephalosporin use also decreased immediately, indicating that the reduction in PIPC/TAZ use was not accompanied by a compensatory increase in the use of this class. Because TORA specifically targeted PIPC/TAZ, however, the decrease in antipseudomonal cephalosporin use cannot be attributed to TORA and may have reflected concurrent changes in prescribing practice, patient case mix, or other unmeasured factors. No concurrent formulary changes or supply restrictions affecting antipseudomonal cephalosporins were identified. The reasons for the different temporal patterns across antibiotic classes could not be determined because patient-level indications, switching patterns, and prescribing appropriateness were not assessed.

The secondary outcomes add clinical interpretability to the utilization findings. The reduction in treatment episodes lasting ≥14 days is consistent with the overall aim of TORA to prompt reassessment of prolonged therapy, although the study design cannot distinguish the direct and indirect effects of the two reminder thresholds. Because the day-7 reminder was posted only after therapy exceeded 7 consecutive days, the reduction in episodes lasting ≥7 days cannot represent a direct effect of the reminder on the same treatment episode. It may, however, partly reflect broader indirect changes in prescribing behavior after TORA implementation, including earlier reassessment of subsequent PIPC/TAZ courses, although these mechanisms were not measured. Combining day-7/day-14 reminders with a 48–72-hour time-out may provide complementary opportunities to address both early antibiotic selection and prolonged treatment and warrants future evaluation. Thirty- and 90-day mortality were similar before and after implementation. In the blood-culture-positive subgroup, 30-day mortality was also not significantly different, although it was higher in the postintervention period than in the preintervention period (12.1% vs 9.5%). However, these comparisons were exploratory, unadjusted descriptive analyses, and the subgroup also included a clinically heterogeneous population. Therefore, this finding should be interpreted cautiously and does not provide definitive evidence regarding patient safety.

An important implication of this study is practical applicability. PAF and preauthorization remain core stewardship strategies, but both are resource intensive and can be difficult to sustain across all departments when stewardship personnel are limited.^1,2^ In contrast, TORA was embedded into routine weekday workflow, required only standardized EMR documentation and a patient-level EMR message-board reminder, and was delivered by rotating ASP pharmacists in approximately 15 minutes per day. Candidate identification and initial filtering were largely automated, whereas final eligibility confirmation required pharmacist review. The transferable components of TORA are the predefined treatment-duration milestones and standardized prompts for reassessment, whereas the EMR extraction, filtering process, reminder interface, and staffing workflow are institution-specific and require adaptation to local resources. Further EMR-based automation could reduce manual workload and facilitate more timely implementation, including on weekends, although the incremental effect of these approaches requires evaluation. Thus, TORA may offer a pragmatic, adaptable complement to more personnel-intensive ASP activities, particularly where direct case-by-case review of all broad-spectrum antibiotic use is not feasible.^2,14^

This study has several limitations. First, it was conducted at a single tertiary-care hospital, which may limit generalizability, although the intervention was implemented across a broad range of inpatient departments other than hematology and transplant surgery. Second, the primary analysis used a single-group ITS design without a concurrent control group, limiting causal attribution to TORA. Although the models included calendar-month fixed effects to account for seasonality, unmeasured concurrent changes may still have influenced the observed associations. In particular, TORA implementation occurred shortly after the peak of the Delta variant–driven fifth wave of coronavirus disease 2019 in Japan, and pandemic-related changes in admission volume, surgical activity, patient severity, or case mix may have contributed to the observed immediate change. In addition, we did not systematically ascertain department-specific changes in antimicrobial-use protocols or clinical practice during the study period, although no concurrent hospitalwide antimicrobial-use policy changes were identified. Nevertheless, the concurrent reduction in prolonged PIPC/TAZ treatment episodes, particularly episodes lasting ≥14 days, and the absence of identified major PIPC/TAZ supply restrictions, formulary changes, or administration-restriction policy changes suggest that TORA may have contributed to the observed reduction. In addition, because aggregated J-SIPHE data included PIPC/TAZ use among patients with infectious diseases consultation who were not eligible for TORA reminders, the observed intervention-associated change may have been attenuated, although the magnitude of this effect could not be determined. Third, the secondary outcomes were exploratory, unadjusted prepost comparisons, and postdischarge deaths occurring outside our healthcare system may not have been completely captured. Fourth, we did not evaluate patient-level antibiotic switching or prescribing appropriateness, use of non-antipseudomonal agents, or oral step-down therapy, including oral fluoroquinolones. Thus, substitution outside the selected balancing outcomes could not be fully assessed, and reduced PIPC/TAZ exposure could reflect discontinuation, de-escalation, intravenous-to-oral switching, or changes in case mix. Despite these limitations, the main findings appear robust given the relatively long observation period, the absence of identified major PIPC/TAZ-specific operational changes, and the consistency between the reduction in prolonged PIPC/TAZ exposure and the intended mechanism of TORA.

In conclusion, TORA was associated with an immediate reduction in inpatient PIPC/TAZ use in targeted departments. Prolonged PIPC/TAZ exposure also decreased, without evidence of major substitution to carbapenems or quinolones. Thirty- and 90-day mortality did not differ significantly between periods, although these comparisons were exploratory and unadjusted, and the study was not designed or powered to detect mortality differences. Given its low implementation burden, this approach may offer a pragmatic stewardship option that can be adapted to local infrastructure and staffing.

## Supporting information

10.1017/ash.2026.10899.sm001 Suzuki et al. supplementary material Suzuki et al. supplementary material

## Data Availability

The data that support the findings of this study are not publicly available because of privacy and ethical restrictions but may be available from the corresponding author upon reasonable request and with appropriate institutional approval.

## Supplementary material

The supplementary material for this article can be found at https://doi.org/10.1017/ash.2026.10899.

## Table 1. Long description

A table with four rows and three columns comparing the effects of TORA implementation on different drug groups. The columns are labeled Drug group, Variable, Estimate (95% CI), and P value. The rows are labeled with different drug groups and variables. Row 1: Drug group: PIPC/TAZ, Variable: Immediate change, Estimate (95% CI): -1.021 (-1.783 to -0.259), P value: .010. Row 2: Drug group: PIPC/TAZ, Variable: Slope change, Estimate (95% CI): -0.007 (-0.044 to 0.031), P value: .724. Row 3: Drug group: Carbapenems, Variable: Immediate change, Estimate (95% CI): 0.109 (-0.354 to 0.572), P value: .639. Row 4: Drug group: Carbapenems, Variable: Slope change, Estimate (95% CI): -0.010 (-0.033 to 0.013), P value: .400. Row 5: Drug group: Antipseudomonal cephalosporins, Variable: Immediate change, Estimate (95% CI): -0.480 (-0.830 to -0.129), P value: .008. Row 6: Drug group: Antipseudomonal cephalosporins, Variable: Slope change, Estimate (95% CI): 0.012 (-0.005 to 0.029), P value: .160. Row 7: Drug group: Quinolones, Variable: Immediate change, Estimate (95% CI): -0.012 (-0.252 to 0.228), P value: .921. Row 8: Drug group: Quinolones, Variable: Slope change, Estimate (95% CI): -0.008 (-0.020 to 0.004), P value: .207.

Navigate back to Table 1..

## Table 2. Long description

A table with four rows and three columns comparing secondary outcomes before and after an intervention. The table is divided into three sections: Treatment-episode-level outcomes, Patient-level outcomes, and Blood-culture-positive subgroup. Column headers are Preintervention, Postintervention, and P value. Row labels include PIPC/TAZ treatment episodes, Episodes lasting ≥7 consecutive days, Episodes lasting ≥14 consecutive days, Patients receiving PIPC/TAZ, 30-day mortality from PIPC/TAZ initiation, 90-day mortality from PIPC/TAZ initiation, Patients with ≥1 positive blood culture within 48 hours before PIPC/TAZ initiation, and 30-day mortality from blood culture collection. Notable trends include a decrease in episodes lasting 7 or more consecutive days and 14 or more consecutive days postintervention. 30-day and 90-day mortality rates remain relatively unchanged. Among patients with positive blood cultures, 30-day mortality slightly increased postintervention.

Navigate back to Table 2..
