# Supplementary material for: Effect of a 7-Day and 14-Day Antibiotic Time-Out Reminder Alert (TORA) on Inpatient Piperacillin-Tazobactam Use: an interrupted time-series study
Source: Antimicrob Steward Healthc Epidemiol. 2026 Oct 5;6(1):e314. doi: 10.1017/ash.2026.10899 (PMC13760303; doi:10.1017/ash.2026.10899)
Supplement: Supplementary file 1 [file S2732494X26108997sup001.docx]

**Antimicrobial Stewardship & Healthcare Epidemiology**

**Supplementary Appendix 1**

**Article title:**

**Effect of a 7-Day and 14-Day Antibiotic Time-Out Reminder Alert (TORA) on Inpatient Piperacillin-Tazobactam Use: An Interrupted Time-Series Study**

**Authors:** Yuhei Suzuki, B.Pharm^1^; Ryo Yamaguchi, PhD^1^; Takehito Yamamoto, PhD^1^; Sohei Harada, MD, PhD^2^; Takehiro Tanaka, B.Pharm^1^; Satoshi Kitaura, MD, PhD^3^; Shinya Matsumoto, MD, PhD^4^; Ryu Yoneda, MD^4^; Takeya Tsutsumi, MD, PhD^3,4^; Tappei Takada, PhD^1^

**Supplementary Appendix 1 title:**

**English translations of the standardized day-7 and day-14 TORA reminder texts**

**Supplementary Appendix 1. English translations of the standardized day-7 and day-14 TORA reminder texts**

The following standardized reminder texts were used for both (1) the brief note in the electronic medical record (EMR) and (2) the patient-level message-board reminder within the EMR.

**Day-7 reminder**

More than 7 days have passed since the initiation of piperacillin-tazobactam (PIPC/TAZ).

This exceeds the usual treatment duration for common infections such as pneumonia, urinary tract infection, and intra-abdominal infection with adequate source control. Please consider whether therapy can be discontinued.

Longer treatment may be indicated for abscess-forming infections, bloodstream infection due to Staphylococcus aureus or Candida species, bone and joint infections, device-related infections, endovascular infections or infective endocarditis, and infections in immunocompromised patients, such as those with neutropenia or receiving immunosuppressive therapy. Please assess these cases individually.

Antimicrobial Stewardship Team (AST)

**Day-14 reminder**

More than 14 days have passed since the initiation of piperacillin-tazobactam (PIPC/TAZ).

This exceeds the usual treatment duration for common infections. Please consider the timing of treatment discontinuation.

If continuation is necessary, please document the reason in the medical record (eg, difficult-to-drain abscess-forming infection, bone and joint infection, device-related infection, endovascular infection or infective endocarditis, or infection in an immunocompromised patient, such as one with neutropenia or receiving immunosuppressive therapy). If the appropriate stop date is difficult to determine, please consider consultation with the infectious diseases service.

Antimicrobial Stewardship Team (AST)
